# Remarks on Spinal Abscesses

**Published:** 1885-12

**Authors:** B. E. Hadra

**Affiliations:** Austin, late of San Antonio, Texas


### Medical ★ Journal,

PUBLISHED MONTHLY AT

AUSTIN, TEXAS.

Vol. 1.] DECEMBER, 1885. [No. 6.

*"Scribimus indocti, doctique!"*

### Original Articles.

☞ Contributed Exclusively to this Journal. ☜

*The Articles in this Department are accepted, and published with the understanding that we are not responsible for, nor do we indorse the views and opinions of the writers, by so doing.*

### REMARKS ON SPINAL ABSCESSES.

*By B. E. Hadra, M. D., Austin, late of San Antonio, Texas.*

Read before the West Texas Medical Association September, 1885,
(For Daniel's Texas Medical Journal.)

ONE after another of the many old surgical dogmas we see fall, and new views, new prospects develope before us, since we have become acquainted with our greatest enemy, and learned how to successfully combat him.

In reviewing the progress of surgery during the last twenty years, since the advent of antisepsis and asepsis, we find that many additions have been made to our stock of knowledge; while so radically have views—once accepted as orthodox—been changed, that we might say a new era has dawned in surgical practice,—one of immense progress. There has been, indeed, a great revolution, with great gain and great successes; a period which only later generations will fully appreciate in the extent of its magnitude.

One of the new additions to active surgery forms the treatment of so-called "cold abscesses." Of these, however, I propose now to speak only of one variety—spinal abscesses.

The literature on the subject is meagre, and unsatisfactory, and heretofore authors have seemed to hold them in a kind of awe. Thus we find even in the latest editions of Holmes' Surgery a distinct warning against operative interference, and a gloomy and frightening picture is given of an opened spinal abscess. We breathe a little easier when we read in Bryant, "spinal abscesses should not be opened hastily. When steadily progressing, however, they must be dealt with, and the best method is, without doubt, to make a free opening," etc.

But then comes * Volkman, the great German bone surgeon, who tells us that spinal abscesses should and must be opened.

I confess that the want of unanimity on the part of the authors on this important point leaves the general practitioner in an uncomfortable position, one of doubt and uncertainty, which compells him to work out the problem for himself in each case, and by a close investigation of the pathology of the disease we may arrive at a tolerably good working basis.

It is not my intention to go into the minutiæ of the pathology of spinal abscesses in this paper; for this you are referred to standard works on the subject. I only wish to call your attention to the fact that almost without exception, in cases of carious vertebral diseases, *tubercle-bacilli* have been found by reliable investigators, and that in consequence of it, the most advanced surgeons in Germany, do not hesitate to treat spondilitis as a *local tuberculosis*, a position also taken by Gross and other eminent surgeons in America.

If it is correct to consider every pathological process in which the bacilli tuberculosis are found, as of true tuberculous nature (and this position is now very generally accepted) of course we are bound to believe such localized tuberculous changes to be common in surgical diseases, which formerly were mistaken for other diseases, of different character and origin. Holding

* Vide Trans German Surgical Society, 1885.

this view, Volkmann enumerates as localized tuberculoses, for instance, absceding glands, lupus, some fistulæ in ano, spina ventosa, tumor albus, most of what was formerly called caries of the bones,* etc. It is at once seen that such doctrine is calculated to overthrow a great many of our old theories; but at the same time it appears prophetic,—a shining light which, dispelling the darkness, shows us firm ground on which we can securely stand and fight advantageously the devouring monster.

If we can accept the doctrine of a localized virus in some part of the human system; if it can exist limited and shut up within the surrounding tissue—we should not hesitate to remove that part if it can be done. The great difference between such an hypothesis and the old humoral pathology is at once to be seen, and it would be satisfactory to know, or at least to believe that the knife can exsect a diseased part of a man's body, like removing the rotten part of an apple; but unfortunately there is a serious objection to such a theory to be found in the unity of the circulation of the fluids of the system, blood and lymph. Pathologists, though, tell us that lymphatic glands act as sieves, and thus protect, partially, the general circulation from certain deleterious elements. We further know from experience that sometimes coarser substances retain their places in the meshes of the different structures, and in addition to that, Volkmann points out the formation of firm membranes (containing largely tubercle bacilli) which nevertheless form a barrier against the further invasion of the poison, and thus protect adjacent organs and tissues—a kind of self-limitation, as it were. In short, there is no doubt that local tuberculosis exists, and that it can exist for an indefinite period, without becoming a general systemic affection.

But let us return to our theme. It is difficult to understand in the first place, how the bacilli obtained access to, and why they located on such an obscure, well protected and secluded place as the vertebræ. Sayres, of New York, was the first, I believe, to claim traumatism as an origin or starting point for all the class of joint diseases represented by coxitis and spondilitis. Volkmann, agreeing with him, explains the formation

* See also report on Surgery by E. J. Beall, M. D. Transactions Texas State Medical Association, 1885.

of tuberculosis thus: We have to assume that the bacillus is easily received into the system, being so very common amongst mankind. Now, so long as the system is strong, and the activity of the tissues is unimpaired, the foe does not get a chance to settle and to thrive; he is overpowered by the normal processes of human life. But nothing seems to be more favorable to prepare the ground for this seed than a traumatism of slight nature, such as blows or falls, which produce a kind of subacute inflammation; while graver injuries set up a more active inflammatory process which seems to overpower and destroy the bacillus. No doubt the every-day's experience of the practitioner will confirm this statement. Thus we have a kind of explanation of the origin of localized tuberculosis in a vertebra, a bone so much exposed to the influence of lighter injuries, which, to say the most of it, is barely better than no explanation.

My remarks thus far, are by no means of a purely academic interest; they are designed to map out a basis for surgical interference; for the time is past when the surgeon was the eminently and exclusively practical man, guided alone by the light of experience, and manual dexterity. We want guiding ideas, and leading theories, deduced from strict and scrutinous recearches. Surgery to-day is more in accord with physiology and pathology, and all the theoretical branches of medicine than ever before, and it is to-day more difficult to draw the line between surgery and medicine than ever.

The practical deduction we have to draw from the premises set forth above is, that it is the duty of the surgeon to *remove the focus of infection* as soon and as thoroughly as circumstances will permit. If caries of the bone is localized tuberculosis, and if tuberculosis is caused by the tubercle-bacilli, evidently we should remove the parasite; or, if this cannot be done, destroy it, or at least impair its vitality and growth, as the case may be. In order to do this we must remove the diseased bone, which is best, where possible; or, if, for anatomical or biological reasons this cannot be done, we must get at the seat as best we can, so as to destroy the seeds, (disinfection) or remove it as much as possible, (drainage). Our action then, is clearly prescribed by our theoretical views.

Now, the first propositon, the removal of the bone is, in caries of the vertebræ, as we all know, most generally a surgical imposibility. It has been attempted; but as the caries is usually in the body of the vertebra, it is only under very extraordinary circumstances that the surgeon is justified it making the attempt at excision. But there are cases in which the spinous processes are the seats of the disease, and there is, as a general thing, no valid objection to their removal to a considerable extent, at least, when easily accessible.

I remember a case of a young lady, who for years under my treatment, underwent many operations for opening and draining spinal abscesses, which formed in great numbers, everywhere, in front, on both sides of the diseased lumbar vertebra, and which had reduced her so much that no one expected her to recover. At one time a spinous process became actively inflamed,—the symptoms were urgent and demanded relief. I cut down and evacuated a quantity of pus and removed some loose spiculæ of bone. There were no evil consequences followed this operation, and under iodoform dressing the wound healed. When removed from Texas to her home in Alabama her case was considered hopeless; but her medical attendant continued the same treatment, opening and draining the abscesses as they formed, and at present she enjoys life as much as formerly,having fully recovered.

I embrace in the first indication for treatment the removal of diseased bones and all loose pieces, (and these latter will often be found in great numbers in old abscesses—generally far away from their original locality, erratic carriers of the bacillus). The second and third indications are drainage and disinfection, or, rather, *germicide.* If there is soundness in these two surgical principles, they are as applicable to spinal abscesses as to other affections. There are two forms of spinal abscess; 1st, such as persist after the original disease has exhausted itself or been eradicated—disconnected sacs filled with pus and spiculæ of bone; (of course containing the tubercle bacilli), and 2nd, those which are still connected with the original fountain, and form *ponds* in the stream running from the spring or source. I suppose no surgeon, however he might dread to touch a cold abscess, would hesitate to make a free incision into these first mentioned, and to establish drainage.

The absence of tenderness on pressure over the region of the spine, in cases of caries of the vertebra, indicates the existence of such an abscess disconnected with the original spinal disease. In such case spiculæ of bone will most likely be found; they act as constant sources of irritation, and will keep up suppuration in the cellular tissue between the muscles. But the pus by itself is capable of acting as a foreign body, being shut in by the pyogenic membrane and remaining unabsorbed. It is clearly the surgeon's duty in such cases to make a free incision and to remove all the contents of the sac, especially bone particles, which are sometimes hard to find. In order to be sure that you have thoroughly removed all foreign matter, it is necessary to make a thorough digital exploration. The removal of all contents being accomplished, the membrane itself must be removed. This is best done by meams of a pledget of cotton carried on a dressing forceps and thoroughly and roughly swabbed around in the cavity. The cavity should then be thoroughly washed out with an an antiseptic wash, and again swabbed, this time with the cotton dusted with iodoform, which is to be well rubbed into the walls. Be certain that your incision is sufficiently large to entirely evacuate the sac, and to enable you to see and examine the whole diseased surface and to prevent premature closure—such were the steps employed in the following case :

Lilly R æt 11 years : When two years of age this child presented evidences of Potts' disease. At six years, spinal disease was diagnosed, but owing to some friendly (?) extra-medical advice, the physician was dismissed. At that time there was swelling over the anterior superior spinous process of the left ilium. It was an abscess, and was steadily growing, and the child speedily became hectic and finally a physical wreck. When I saw her there was a gibbus in the lumbar portion of the vertebral column, but not the slightest tenderness over it, upon pressure. In front, over the left crest of the ilium there was a bluish fluctuating tumor as large as a child's head. With the assistance of Dr. Kingsley, this tumor was freely incised, a great mass of flocculent pus and several loose pieces of bone were removed. The cavity was then well irrigated and rubbed out with iodoform in the manner above described. There was no connection to be detected with any deeper structure. The child made a rapid recovery, becoming a healthy and happy creature in the remarkably short time of two weeks, by which time union was perfect.

But in cases where caries of one or more of the vertebrae still exists, and the abscess is in direct communication with it, it is quite different, and we can not hope for such results in so short a space of time. The question of opening is of much greater importance, because experience has shown that active operative interference has, in many cases, been attended with harm; still, in pursuance of the views above set forth, we must incise them, as offering the best chance for recovery. It is the only way, indeed, in which the localized tuberculosis can be exterminated, and prevented from further extension, It must be, that if the operation formerly, by surgeons who believe in Listerism, was not so successful as at the present time, their mode of procedure, and after treatment, were not correct. In the light of recent experience, to bring a closed and protected abscess into communication with the atmosphere without antiseptic care, or to use unclean instruments is but to kindle a dormant fire. The strictest antiseptic precautions should be employed, not only in opening, but in the after treatment of such abscesses. Aspiration is practically valueless except for diagnostic purposes; it will not do in lieu of a free incision.

But in small abscesses, and where the general health is not suffering, there being neither fever, nor pain, it would hardly be justifiable to make an incision, and thus incur all the care and troubles entailed by a thorough antiseptic course of after treatment; it is only when the case is steadily progressing and the symptoms become more urgent, that we should operate.

In accordance with this doctrine, therefore, I treated the following two cases.

CASE 1.—M. D., girl two years of age, daughter of healthy parents, in good circumstances, began complaining nine months ago, as children do in the beginning of coxitis—loss of appetite, hectic fever, flexion of one leg, inability to stand erect, etc. So much did the case resemble hip-joint disease, that one of the best known surgeons in Texas pronounced it such. Still there was not the slightest pain in moving the leg at the hip-joint. A plaster bandage was applied, embracing the pelvis and extending on the diseased side to the knee-joint. This was removed six weeks later, and to my surprise, an abscess was found just over the brim of the left ilium—bulging far out. Further examination revealed a slight prominence of the 3rd and 4th lumbar vertebrae, which were painful on pressure. Our mistake in diagnosis was then evident. The abscess was freely opened—of course under strict antiseptic precaution—the finger introduced and carrid easily to the diseased bone. Several small spiculae of necrotic bone were removed along with the pus; the cavity was well washed out and iodoform introduced on a pledget of cotton at the end of a sound. This was well rubbed in, up to the bone. A Stillman's brace was applied and the child improved rapidly, and under careful antiseptic attention, drainage and washing, did well. The wound remained open, and after three months, the progress towards final recovery was still not satisfactory,and another search was instituted. This time the finger was not able to reach to the bone, the cavity having very much contracted, and though I expected to find a piece of bone, I did not, but instead, a large pyogenic membrane came away in rubbing with the cotton; treatment as before. The child again improved and apparently was restored to perfect health; the abscess closed, appetite and strength returned. Still, after awhile, the wound opened somewhat, though the general health is at this time excellent. I expect at another examination, to find a little spicula of bone, or that it will come away.

CASE 2.—K. E., 8 years old. Three years ago the girl fell on her head. No untoward symptoms developed immediately, but in a short time she began to complain of pain in her chest. Three months later she carried her head bent forward and could not take a deep inspiration. Hiccough became very annoying, She was sent to Indianapolis, where braces were applied to keep the head back. For about eighteen months there has been an abscess on the neck, which had a strange behavior; it forms a kind of blister and bursts about every 3rd day, discharging pus freely. Her general health is feeble, but tolerable under aid of the braces. When I first saw the child I found her pale, walking with the aid of a heavy, but well sustaining machine, the head bent forward, and a hump on the junction of the first thoracic and last cervical vertebra, forming an angle between the cervical and thoracic column of about 110 degrees. According to the statement of her relatives, her condition has greatly improved since she went to Indianapolis. On the left side of the neck, external to the sterno cleido mastoideus—sternal portion, there was a fistulous opening. Under chloroform, a sound was inserted, and after some manipulating a fistulous canal was detected leading down to rough, diseased bone. The sound was then armed with cotton, and this well filled with iodoform, was again introduced, with the intention of inserting as much of the iodoform as the cotton would convey. A decided improvement followed—in her general health, as well as in the diminished secretion of pus. The pus did not re-accumulate, though a slight discharge constantly took place. In three weeks the operation was repeated, the case being still under my care.

In conclusion I will state that I make no claim whatever for originality, my paper being intended as a review or survey of the modern theories and opinions on this important, but often neglected, or overlooked disease; but I do claim it to contain correct deductions from the teachings of the best authorities of to-day. I have only given cases which were treated in accordance with the new doctrines—some being still under treatment and not yet terminated.

Post Script.—Since the above was written I have seen in the *Journal* of the *American Medical Association* of Nov. 7, a paper by Dr. Ed. Andrews, on Lumbar Abscess, wherein he expresses some views so fully in accord with those above given—though differing in other respects, that I beg to call the readers attention to it.

